# MicroRNA-125b promotes tumor metastasis through targeting tumor protein 53-induced nuclear protein 1 in patients with non-small-cell lung cancer

**DOI:** 10.1186/s12935-015-0233-x

**Published:** 2015-09-17

**Authors:** Qinchuan Li, Yang Han, Chunhong Wang, Shan Shan, Yuanyuan Wang, Jingang Zhang, Tao Ren

**Affiliations:** Department of Cardiothoracic Surgery, East Hospital, Tongji University School of Medicine, Shanghai, China; Department of Pathology, East Hospital, Tongji University School of Medicine, Shanghai, China; Department of Respiratory Medicine, East Hospital, Tongji University School of Medicine, 150 Jimo Road, Pudong New Area, 200120 Shanghai, China; Service Center for Family planning, Maternal and Child Health Care, Lanshan, Linyi, Shandong China

**Keywords:** NSCLC, Metastasis, miR-125b, TP53INP1

## Abstract

**Background:**

Lung cancer, predominantly non-small-cell lung cancer (NSCLC), is the leading cause of cancer deaths worldwide. There is a great need to identify critical effectors involved in metastasis of NSCLC that will facilitate the development of new therapeutic strategies. Here we evaluated the potential role of miR-125b in the metastasis of NSCLC cells.

**Methods:**

Human NSCLC cells were isolated from surgical tissues with Cancer Cell Isolation Kit. Expressions of miR-125b and TP53INP1 were detected with real-time PCR and western blot. Human miR-125b mimics, miR-125b inhibitor, TP53INP1 expression plasmid and TP53INP1 siRNA were transfected into NSCLC cells with nucleofector transfection kit. NSCLC metastasis was determined with adhesion assay, invasive assay and lung tumor metastasis model.

**Results:**

The expression of miR-125b was significantly higher in poorly differentiated NSCLC cells that are endowed with high metastatic potentials. Up-regulation of miR-125b could enhance the metastatic potential of NSCLC cells in vitro and in vivo, while down-regulation of miR-125b resulted in decreased metastatic potentials in vitro and in vivo. Further, tumor protein 53-induced nuclear protein 1 (TP53INP1) was an important target of miR-125b involved in metastasis of NSCLC cells. TP53INP1 served as a negative regulator of NSCLC metastasis. Decreased expression of TP53INP1 in tumor tissues was inversely associated with their expression of miR-125b, significantly lower in poorly differentiated tumors and inversely correlated with the clinical stages in patients with NSCLC.

**Conclusions:**

These findings demonstrated that miR-125b promoted tumor metastasis via targeting TP53INP1 in human NSCLC cells, which uncovered a real clinical relevance of microRNAs in tumor biology, and provided novel potential candidates for NSCLC clinical practice.

**Electronic supplementary material:**

The online version of this article (doi:10.1186/s12935-015-0233-x) contains supplementary material, which is available to authorized users.

## Background

Lung cancer, especially non-small-cell lung cancer (NSCLC), is the most common cause of human cancer related mortality [1–4]. Although combined treatments including surgery, chemotherapy, radiotherapy and targeted therapy were applied in clinical management, the current outcome of NSCLC patients is still far from satisfied, which is mostly because of NSCLC metastasis [4–7]. Therefore, exploration of critical effectors involved in NSCLC metastasis is urgently needed.

MicroRNAs (miRNAs) are non-coding RNAs and regulate target genes at post-transcriptional level [8, 9]. It is well acknowledged that deregulation of miRNAs was involved in tumor initiation and progression [10]. MiR-125b, a human homologue of lin-4, was reported to be involved in tumor progression [11–15]. MiR-125b could regulate tumor cellular apoptosis and proliferation [16–21]. In NSCLC, serum miR-125b was significantly increased and was positively associated with NSCLC stages and poor patient survival [9, 10]. Of note, miR-125b expression in poorly differentiated NSCLC was significantly higher than those in well and moderately differentiated NSCLC [9, 10]. These findings indicated an involvement of miR-125b in NSCLC metastasis, which is largely undefined.

In this study, we evaluated the effect of miR-125b on metastasis of lung cancer cells from NSCLC patients. We observed a significant higher expression level of miR-125b in poorly differentiated NSCLC cells. Of important, management of miR-125b expression could modulate the NSCLC metastasis in vitro and in vivo. Further, tumor protein 53-induced nuclear protein 1 (TP53INP1) was identified as a critical target of miR-125b involved in NSCLC metastasis. Our findings provided new insights into the function of miR-125b during the metastasis of human NSCLC and were helpful for developing novel strategy in treatment of NSCLC patients.

## Results

### High expression of miR-125b in poorly differentiated human NSCLC

**Fig. 1:**
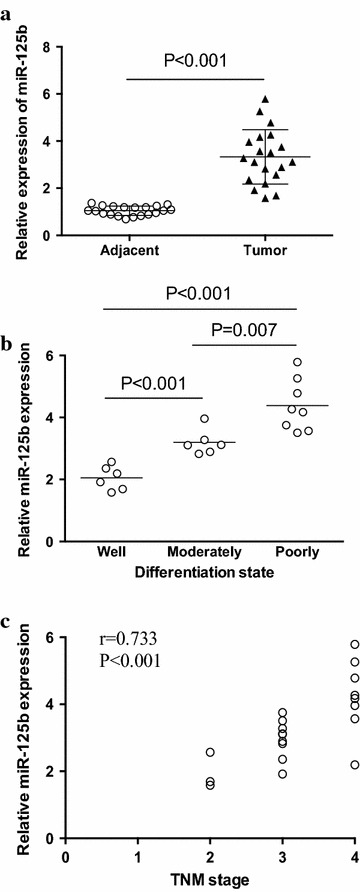
Expression of miR-125b in human NSCLC cells. **a** Relative expression of miR-125b was determined by real time PCR in 20 NSCLC lung cancer samples. **b** The relative expression of miR-125b in the well, moderately and poorly differentiated tumor tissues were shown. **c** The correlation between the relative miR-125b expression and the TNM stage of NSCLC patients was analyzed. *Each dot* represented the results from one patient

Detection of miR-125b expression in clinical NSCLC patients showed that the expression level of miR-125b was higher in lung cancer tissue compared with adjacent tissue (Fig. 1a). We further showed that the level of miR-125b was significantly higher in poorly differentiated NSCLC than those in well and moderately differentiated cancers (Fig. 1b). Besides, miR-125b expression was positively correlated with the clinical stages of patients with NSCLC (Fig. 1c). These findings suggested that miR-125b might be involved in NSCLC metastasis of clinical patients.

### Up-regulation of miR-125b promoted human NSCLC metastasis

**Fig. 2:**
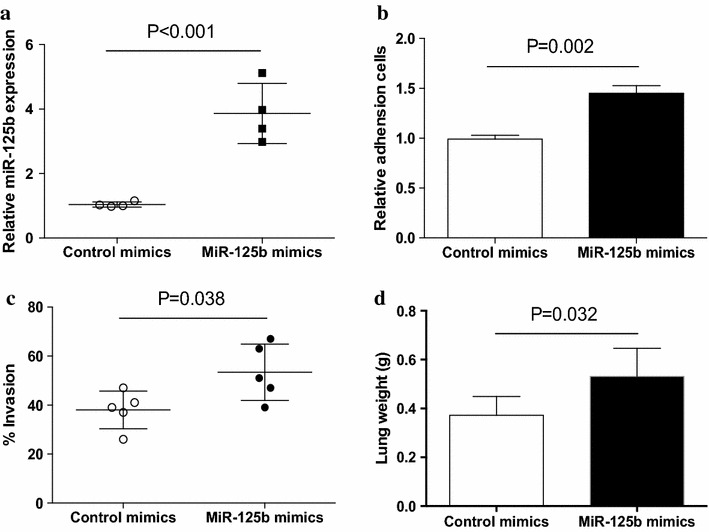
Up-regulation of miR-125b enhanced NSCLC metastasis. **a** NSCLC cells from different patients were transfected with miR-125b mimics for 12 h and then assayed for their expressions of miR-125b. *Each dot* represented the results from one patient. **b**, **c** NSCLC cells from different patients were transfected with miR-125b mimics or the control, respectively, and then assayed for their adhesion activity (n = 4) and invasion (n = 5). **d** Nude mice were challenged LPS and NSCLC cells that were transfected with miR-125b mimics or the control. Lung tumor burden was detected by analyzing lung weight. Data were presented as means (±SD) from five nude mice in each group

To determine the effect of miR-125b on metastatic potential of human NSCLC, NSCLC cells were transfected with miR-125b mimics and then analyzed for their metastatic potential. As shown in Fig. 2a, transfection with miR-125b mimics effectively enhanced its expression level in NSCLC cells (p < 0.05). Given that adhesion of tumor cells to extra-cellular matrix and basement membranes are considered to be the initial step in the invasive process for metastatic tumor cells, NSCLC cells transfected with miR-125b mimics were examined for their adhesion activities to the substrates precoated with fibronectin, which is a basement member component. We found that transfection with miR-125b mimics significantly elevated the adhesion activities of NSCLC cells (Fig. 2b). Then, we analyzed the invasive potential of NSCLC cells after transfection with miR-125b mimics, and revealed that transfection with miR-125b mimics effectively enhanced the invasion of NSCLC cells (Fig. 2c). To confirm these results in vivo, nude mice were challenged with NSCLC cells that were transfected with miR-125b mimics or control, respectively. Over-expression of miR-125b significantly enhanced the lung tumor burdens of NSCLC cells (Fig. 2d). These results demonstrated that up-regulation of miR-125b could promote the metastatic potential of human NSCLC.

### Down-regulation of miR-125b reduced human NSCLC metastasis

**Fig. 3:**
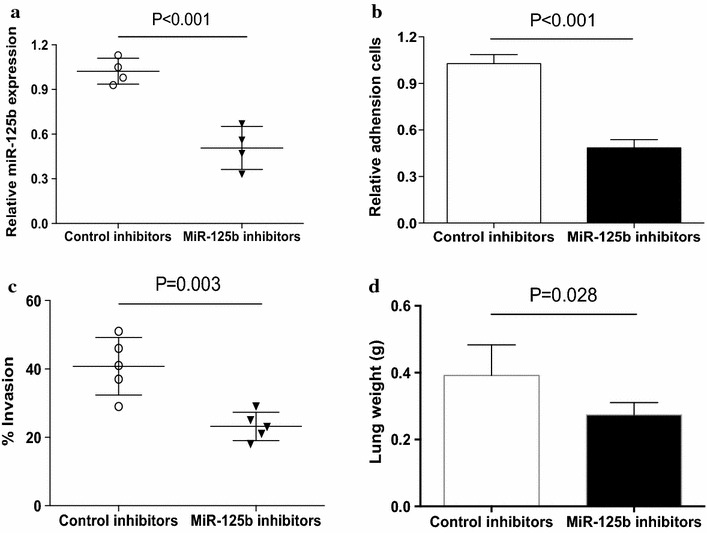
Down-regulation of miR-125b reduced NSCLC metastasis. **a** NSCLC cells from different patients were transfected with miR-125b inhibitors for 12 h and then assayed for their expression of miR-125b. *Each dot* represented the results from one patient. **b**, **c** NSCLC cells from different patients were transfected with miR-125b inhibitors or the control, respectively, and then assayed for their adhesion activity (n = 4) and invasion (n = 5). **d** Nude mice were challenged LPS and NSCLC cells that were transfected with miR-125b inhibitors or the control. Lung tumor burden was detected by analyzing lung weight. Data were presented as means (±SD) from five nude mice in each group

To further elucidate the effect of miR-125b on the metastatic potential of human NSCLC, NSCLC cells were transfected with miR-125b inhibitors and then analyzed for their metastatic potential. As shown in Fig. 3a, transfection with miR-125b inhibitors effectively decreased its expression level in NSCLC cells. Further, transfection with miR-125b inhibitors significantly inhibited the adhesion activities of NSCLC cells (Fig. 3b). Expectedly, transfection with miR-125b inhibitors also effectively abrogated the invasion of NSCLC cells (Fig. 3c). To further confirm this phenomenon in vivo, nude mice were challenged with NSCLC cells that were transfected with miR-125b inhibitors or control, respectively. Decreased expression of miR-125b considerably reduced the lung tumor burdens of NSCLC cells (Fig. 3d). These results demonstrated that down-regulation of miR-125b could inhibit the metastatic potential of human NSCLC.

### TP53INP1 was the dominant target of miR-125b in regulating NSCLC metastasis

**Fig. 4:**
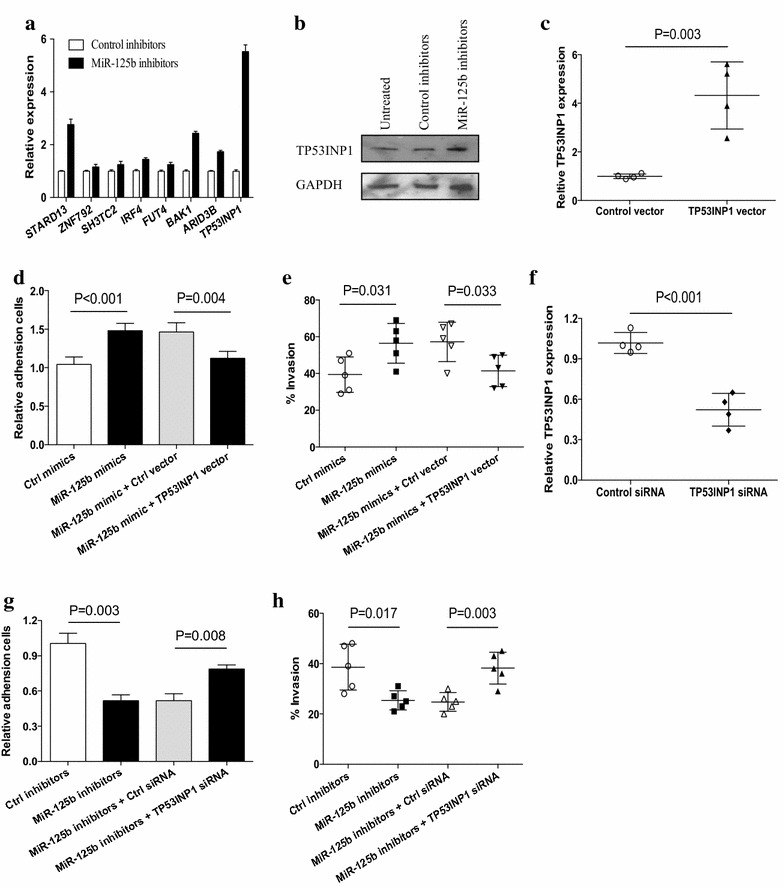
TP53INP1 was a functional target of miR-125b. **a** NSCLC cells from different patients (n = 3) were transfected with miR-125b inhibitors for 12 h and then assayed for their expression of the indicated genes using real time PCR. **b** NSCLC cells from one patient were transfected with miR-125b inhibitors for 48 h and then assayed for their expression of TP53INP1 using western blot. **c** NSCLC cells from different patients were transfected with TP53INP1 expression vector for 12 h and then assayed for their expression of TP53INP1 mRNA level. **d**, **e** NSCLC cells from different patients were co-transfected with miR-125 mimics and TP53INP1 expression vector, and then assayed for their adhesion activity (n = 4) and invasion (n = 5). **f** NSCLC cells from different patients were transfected with TP53INP1 siRNA or the control for 12 h and then assayed for their expression of TP53INP1. **g**, **h** NSCLC cells from different patients were co-transfected with miR-125 inhibitors and TP53INP1 siRNA, and then assayed for their adhesion activity (n = 4) and invasion (n = 5). *Each dot* represented the results from one patient

To further understand the effect of miR-125b on the metastasis of human NSCLC, we predicted the targets of miR-125b by prediction programs including TargetScan and PicTar, and selected eight possible target including STARD13, ZNF792, SH3TC2, IRF4, FUT4, BAK1, ARID3B, and TP53INP1 for real time PCR analysis. We found that the expression of TP53INP1 exhibited a dramatically elevation in NSCLC cells transfected with miR-125b inhibitors (Fig. 4a). To confirm this result, we performed western blot to detect the expression of TP53INP1 in NSCLC cells transfected with miR-125b inhibitors. We found that the protein level of TP53INP1 was significantly increased by transfection with miR-125b inhibitors in NSCLC cells (Fig. 4b). Given that TP53INP1 has been proved to be a direct target of miR-125b [27], we further evaluated the possible role of TP53INP1 in tumor promoting activity of miR-125b. We found that transfection of miR-125b mimics failed to enhance the adhesion activity and invasion of NSCLC cells that were co-transfected with TP53INP1 expression vector (Fig. 4c–e). To confirm this phenomenon, NSCLC cells were co-transfected with TP53INP1 siRNA and miR-125b inhibitors, and then analyzed for their metastatic potential. As shown in Fig. 4f–h, transfection with TP53INP1 siRNA effectively decreased TP53INP1 expression in NSCLC cells and abrogated the effect of miR-125b inhibitors on adhesion activity and invasion of NSCLC cells. These results suggested that TP53INP1 was a bona fide target of miR-125 in regulating the metastasis of human NSCLC.

### TP53INP1 negatively regulated the metastasis of human NSCLC

**Fig. 5:**
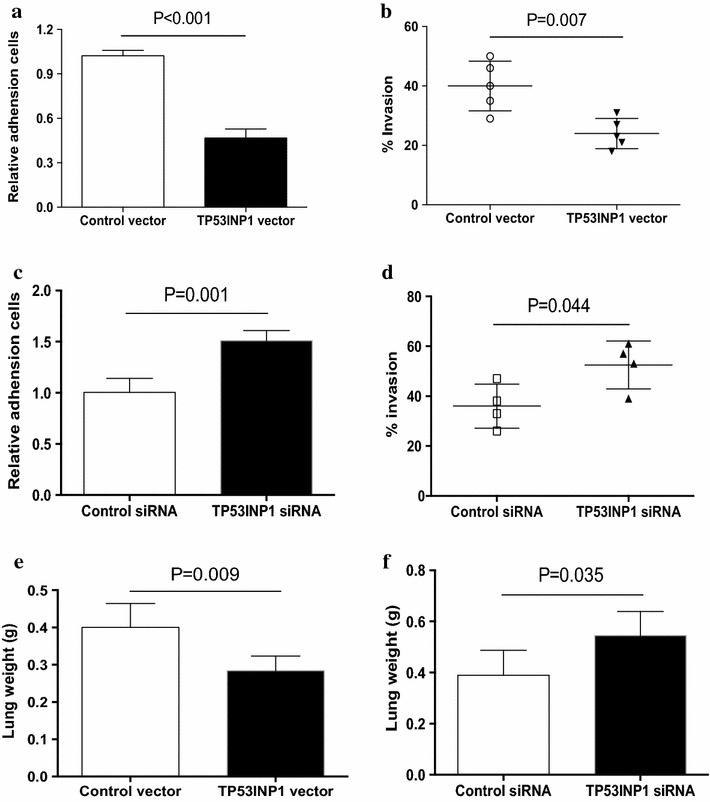
TP53INP1 suppressed NSCLC metastasis. **a**, **b** NSCLC cells from different patients were transfected with TP53INP1 expression vector and then assayed for their adhesion activity (n = 4) and invasion (n = 5). **c**, **d** NSCLC cells from different patients were transfected with TP53INP1 siRNA and then assayed for their adhesion activity (n = 4) and invasion (n = 4). **e**, **f** Nude mice were challenged LPS and NSCLC cells that were transfected with TP53INP1 expression vector, TP53INP1 siRNA or the controls. Lung tumor burden was detected by analyzing lung weight. Data were presented as means (±SD) from five nude mice in each group

We detected the direct effect of TP53INP1 on the metastasis of NSCLC cells. NSCLC cells were transfected with TP53INP1 expression vector and then analyzed for their adhesion activity and invasion. We found that transfection with TP53INP1 expression vector effectively inhibited the adhesion activity and invasion of NSCLC cells (Fig. 5a, b). Transfection with TP53INP1 siRNA significantly increased the adhesion activity and invasive potential of NSCLC cells (Fig. 5c, d). In consistent, enforced TP53INP1 alleviated the lung tumor burden of NSCLC cells, while decreased TP53INP1 enhanced the lung tumor burden of NSCLC cells (Fig. 5e, f). These data suggested that TP53INP1 was a negative regulator of human NSCLC metastasis.

### TP53INP1 expression in tumor tissues was correlated with miR-125b expression and clinical parameters in patients with NSCLC

**Fig. 6:**
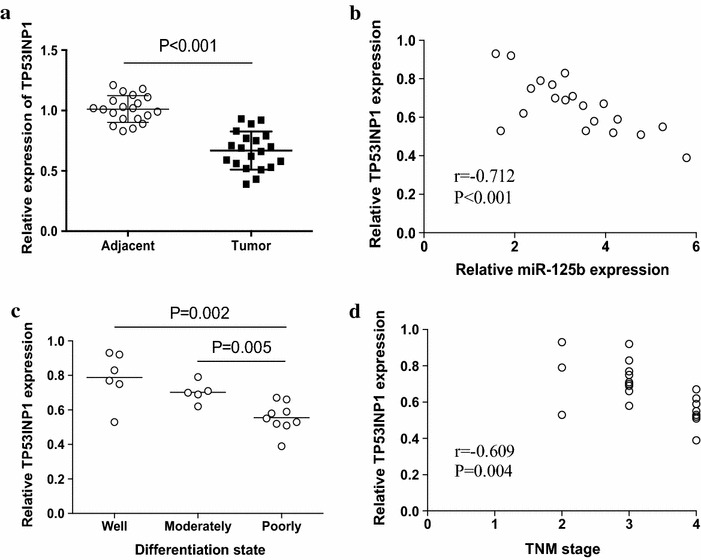
Expression of TP53INP1 in tumor tissues of NSCLC patients. **a** Relative expression of TP53INP1 was determined by real time PCR in 20 NSCLC lung cancer samples. **b** The correlation between the relative miR-125b expression and the relative TP53INP1 expression in NSCLC patients was analyzed. **c** The relative expression of TP53INP1 in the well, moderately and poorly differentiated tumor tissues were shown. **d** The correlation between the relative TP53INP1 expression and the TNM stage of NSCLC patients was analyzed. *Each dot* represented the results from one patient

To further investigate the clinical relevance of our above findings, we detected the relationship between the expression of miR-125b and TP53INP1 in clinical NSCLC patients. As shown in Fig. 6a, the expression of TP53INP1 was significantly lower in tumor tissues compared with adjacent tissues. We further revealed that the expression of miR-125b was inversely correlated with the expression level of TP53INP1 in tumor tissues (Fig. 6b). The expression of TP53INP1 was significantly decreased in poorly differentiated NSCLC than those in well and moderately differentiated cancers, and was inversely associated with the clinical stages of NSCLC patients (Fig. 6c, d). These findings assigned TP53INP1 as an important target for miR-125b in promoting the metastasis of human NSCLC.

## Discussion

Tumor metastasis is the most prominent problem in clinical treatment of cancer, as most cancer mortality is associated with disseminated disease rather than the primary tumor [22]. However, the underlying mechanisms involved in the metastasis of tumor still remain unclear. MiRNAs are known to regulate the expression of genes involved in tumor initiation, proliferation, apoptosis and metastasis [22, 23]. In present study, we reported the critical role of miR-125b in regulating the metastasis of NSCLC. We observed a higher expression level of miR-125b in poorly differentiated NSCLC cells. We validated the elevated expression of miR-125b in tumor tissues and its positive association with the clinical stages. Of important, up-regulation of miR-125b expression enhanced NSCLC metastatic potential in vitro and in vivo, while down-regulation of miR-125b expression decreased their metastatic potentials. Our findings were consistent with recent study which showed that serum miR-125b was significantly increased in NSCLC patients, was positively associated with NSCLC stages and poor patient survival, and was significantly higher in poorly differentiated NSCLC [9, 10]. Our findings could enlarge our understanding of the potential role of miR-125b in NSCLC and suggested that miR-125b was a promising target for treatment of NSCLC.

TP53INP1 is widely recognized as a tumor suppressor gene with anti-proliferative and pro-apoptotic functions [24, 25]. The expression of TP53INP1 was reduced in various human cancers, e.g., breast, pancreas and gastric cancers [26–28]. Of interest, reduction of TP53INP1 expression in gastric cancer was closely correlated with their aggressive phenotypes [28]. These findings suggested TP53INP1 as an important effector in tumor suppression. In this study, we found that TP53INP1 was an important target of miR-125b in regulating the metastasis of NSCLC. We found that over-expression of TP53INP1 could significantly abrogate the tumor promoting effect of miR-125b. Further, up-regulation of TP53INP1 could significantly inhibit the metastasis of NSCLC cells. Decreased expression of TP53INP1 effectively promoted NSCLC metastasis. Finally, we found that the decreased expression of TP53INP1 was inversely correlated with the expression of miR-125b and the clinical stages, and was decreased further in poorly differentiated tumors in patients with NSCLC. In NSCLC cells without treatment, expression of miR-125b was also negatively associated with TP53INP1 expression (Additional file 1: Figure S1). Our findings were, to some extent, in line with recent study which showed that miR-125b could promote proliferation and migration of type II endometrial carcinoma cells through targeting TP53INP1 [29]. However, the precise mechanisms for how TP53INP1 functioned in metastasis of NSCLC undoubtedly deserved successive studies. Besides, the number of patients enrolled in this study was relatively limited and additional study on a large size of clinical samples might substantiate our findings.

## Conclusions

Herein, we reported a critical role of miR-125b in NSCLC metastasis via targeting TP53INP1 in clinical patients. These findings were derived from clinical samples and thus closely reflected the real clinical relevance. MiR-125b and TP53INP1 might be promising targets for developing novel therapeutics for NSCLC clinical practice.

## Methods

### Patients

The human study was approved by the Ethics Committee of Tongji University. Totally 37 NSCLC patients were enrolled in this study and given written informed consent before collecting surgical tissues and clinical parameters. Review of pathology reports confirmed the diagnosis. Subjects with autoimmune diseases or infections were excluded. Information regarding clinical pathological characteristics of patients was summarized in Table 1.

**Table 1:** The clinical pathological characters of the NSCLC patients

Clinical pathological parameter	Number
Sex	
Male	26
Female	11
Age (years)	51–78
Tumor stage	
T1/2	6
T3/4	31
Nodal status	
N0/1	7
N2/3	30
Histological type	
Adenocarcinoma	29
Others	8

Lymph nodal metastasis is according to pathological diagnosis and clinical palpation. Clinical stage is according to TNM stage

### Reagents and cell culture

NSCLC cells were isolated from surgical tumor tissues using Cancer Cell Isolation Kit (Panomics). According to the manufacture’s instructions, cells were cultured for 4 days at 37 °C under 5 % CO_2_ in complete RPMI 1640 medium (GIBCO, containing 10 % heat-inactivated fetal bovine serum supplemented with 2 mM glutamine, 100 IU/ml penicillin and 100 mg/ml streptomycin sulfate), and used for experimental research. MiR-125b mimics and miR-125b inhibitor were from Ribobio (Guangzhou, China). Human TP53INP1 expression plasmid was purchased from Origen. Human TP53INP1 siRNA was from Santa Cruz. The nucleofector transfection kit was purchased from Amaxa.

### Adhesion assay

Cell adhesion assay was assayed as described previously [22, 30]. In brief, microtiter wells were coated with fibronectin (Sigma, St. Louis, MO, USA) overnight and were blocked for 30 min with 0.5 % BSA in PBS. NSCLC cells were suspended at a final concentration of 5 × 10^5^ cells/ml in serum-free medium for seeding. The MTT-assay (Cayman) was used to determine the number of remaining cells (adherent cells).

### Invasive assay

The BD Biocoat Matrigel Invasion Chamber assay was performed as described by the manufacturers (8 μm, BD Bioscience). Briefly, the Matrigel inserts were rehydrated and 5 × 10^4^ NSCLC cells were resuspended in 0.5 mL of serum-free media and then seeded onto the upper chamber of Matrigel-coated filters. In the lower chambers, 0.75 mL of complete medium was added as a chemoattractant. The whole chamber was placed in one well of a 24-well plate, and cells were cultured in routine conditions. After 24 h, the cells on the upper side of the chamber were scraped, and the ones on the lower side of the chamber were fixed by methanol, stained with hematoxylin, and invaded cells were counted under the microscope. Five predetermined fields were counted for each membrane, and the mean values from three independent experiments in triplicates were used. Data are expressed as the percentage of invasion through the Matrigel Matrix and membrane relative to the migration through the control membrane according to the manufacturer’s manual.

### Real-time PCR

Quantitative Real-time RT-PCR was performed as previously described [31, 32]. All the primers and probes were obtained from Applied Biosystems. Total RNA was extracted using TRIzol reagent. cDNA was synthesized with the PrimeScript RT reagent Kit (TaKaRa). Quantitative RT-PCR (qRT-PCR) analyses were carried out to detect mRNA expression using SYBR Premix Ex Taq (TaKaRa), and β-actin was used as an internal control. TaqMan micro-RNA assays (Applied Biosystems) were used to quantitative the expression levels of mature miR-125b, and U6 small nuclear RNA was used as an internal control.

### Western blotting

Cells were lysed with M-PER protein extraction reagent (Pierce) supplemented with a protease inhibitor cocktail. Cytoplasmic and nuclear extracts were prepared using NE-PER nuclear and cytoplasmic extraction reagents (Pierce). After centrifugation at 13,000*g* under 4 °C for 15 min, the supernatants were collected, and the protein concentration of the extracts was measured by BCA Protein Assay (Pierce) according to manufacturer’s instructions. Twenty micrograms of the protein were loaded onto 10 % SDS–polyacrylamide gels and transferred for 90 min at 100 V onto polyvinylidene fluoride membranes using a wet transfer system. The membranes were washed in 5 % skim milk in phosphate buffered saline plus 0.05 % Tween 20 (PBST) for 2 h in order to block nonspecific protein binding sites on the membrane. Immunoblotting was performed using monoclonal antibodies to TP53INP1 and GAPDH (Sigma) at a dilution of 1:1000 in nonfat milk Tris buffer. The membrane was then washed in PBST, probed with a secondary anti-rabbit antibody conjugated to horseradish peroxidase (Amersham Life Sciences) at a dilution of 1:5000, developed using an ECL Western Blotting KIT (Pierce), and exposed to X-ray film (Kodak).

### Lung tumor metastasis model

BALB/c nude mice were purchased from animal center of Tongji University and housed under specific pathogen-free conditions. Lung tumor metastasis model were performed as previously described [33, 34]. Briefly, nude mice (n = 5 per group) were intratracheally challenged with LPS (10 μg/mouse), and injected with 4 × 10^5^ NSCLC cells via tail vein 6 h later. Two weeks later, nude mice were detected for their lung tumor metastases/burdens that were reflected by total lung weights. The mice experiments were approved by Ethics Committee of Tongji University.

### Statistical analyses

T tests and Pearson correlation were used for statistical analyses using the program PRISM 6.0 (GraphPad Software Inc., San Diego, CA, USA). A value of P < 0.05 was considered statistically significant.

## Additional file

**Additional file 1: Figure S1.** Expressions of miR-125b and TP53INP1 in isolated NSCLC cells were determined by qPCR and analyzed for their negative correlation.
