# Supplementary material for: MicroRNA-125b promotes tumor metastasis through targeting tumor protein 53-induced nuclear protein 1 in patients with non-small-cell lung cancer
Source: Cancer Cell Int. 2015 Sep 17;15:84. doi: 10.1186/s12935-015-0233-x (PMC4573481; doi:10.1186/s12935-015-0233-x)
Supplement: Supplementary file 1 — Additional file 1: Figure S1. Expressions of miR-125b and TP53INP1 in isolated NSCLC cells were determined by qPCR and analyzed for their negative correlation. [file 12935_2015_233_MOESM1_ESM.pdf]

Supplementary Figure 1

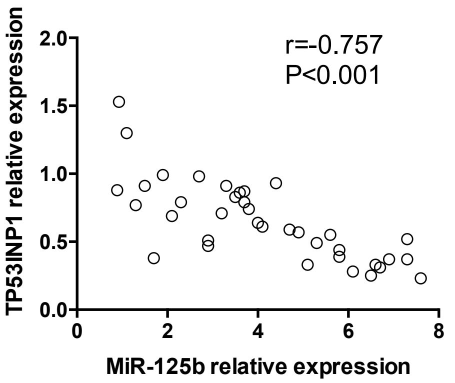

Expressions of miR-125b and TP53INP1 in isolated NSCLC cells were determined by qPCR and analyzed for their negative correlation.
